# Spatio-temporal variations of conservation hotspots based on ecosystem services in Xishuangbanna, Southwest China

**DOI:** 10.1371/journal.pone.0189368

**Published:** 2017-12-12

**Authors:** Shiliang Liu, Yijie Yin, Fangyan Cheng, Xiaoyun Hou, Shikui Dong, Xue Wu

**Affiliations:** School of Environment, Beijing Normal University, Beijing, China; Pacific Northwest National Laboratory, UNITED STATES

## Abstract

Integrating biodiversity and ecosystem services (*BES*) has been viewed as an appropriate approach to identifying conservation priorities. Taking Xishuangbanna tropical region in Southwest China, different *BES*s (habitat quality [used as a proxy for biodiversity], carbon storage, and water yield) were quantified using the InVEST model and conservation hotspots from 1976, 1990, and 2010 were identified by overlapping and ranking the service layers. Results showed that *BES*s areas were unevenly distributed. High habitat quality and carbon storage areas located in the eastern part of the region were mainly occupied by broad-leaved forest, while high water yield areas were covered by grassland and tropical forests. Recognized hotspots were primarily composed of the broad-leaved forest and shrub grassland. However, these habitat types declined by nearly 50% from 1.25×10^5^ ha to 0.63×10^5^ ha and became more fragmented during the study period. We also found that the sub-watersheds which decreased in *BES* had fewer hotspots distributed and suffered greater landscape fragmentation. Our study further explored the impacts of land-use conversion on *BES*, and illustrated the necessity and feasibility of *BESs* in identifying potential conservation areas.

## 1. Introduction

Land conversion from natural forest to agricultural land or built-up land has been reported worldwide in recent decades, putting continuous pressures on local ecosystems and landscapes, especially biodiversity and habitat quality in some tropical regions [1–3]. Previous studies have only focused on biodiversity when planning conservation strategies [4,5]. However, systematic conservation planning should take into account landscape pattern, ecological vulnerability and anthropogenic threat [6]. As biodiversity is a determinant of ecosystem process and plays a key role in many ecosystem services [7,8], both biodiversity and ecosystem services (*BES*) should be modeled to identify hotspots through comparing their spatial patterns [9]. Ecosystem services, bridging natural ecology and the needs of human society, are the rationale for practical applications for environmental sustainability and conservation plans [10–12]. Some strategies have been geared toward combining biodiversity with ecosystems services in terms of comprehensive application in planning sustainable conservation interventions [13,14]. For conservation, the term “hotspot” was originally used for regions only with high species richness for biodiversity conservation [5]. In our study, “hotspot” means sites or areas which have high *BES* values.

Land-use management and planning (reforestation, plantation, agricultural practices, and human activities) can cause variations in the spatial provision of ecosystem services [8,15]. Land-use change (*LUC*) affects the process of local material circulation and energy flow while changing landscape patterns [16]. Decisions about land-use usually aim to maximize a single output such as wood or agricultural production, but such decisions often generate an attendant decline of other ecosystem services [17]. Therefore, it is vital to find out the relationship between *LUC* and biodiversity and ecosystem services change (*BESC*) at a specific scale, and reduce the threats to biodiversity as well as improve regional sustainable development.

Spatially explicit values of ecosystem services that might reflect land-use and management decisions are still not enough [17]. The InVEST (Integrated Valuation of Ecosystem Services and Trade-offs) model provides practical tools to visualize the ecosystem services. As a spatially explicit integrated modeling tool, it is a suite of GIS models and algorithms that converts changes in land-use patterns into changes in ecosystem services [18]. There have been many studies that have used the InVEST model to explicitly elucidate different ecosystem services [8,19], and to analyze as well as forecast ecosystem service dynamics under stakeholder-defined scenarios of land use [3,20]. In China, this model has been applied to the Three Rivers Source Areas, Qinghai province, Poyang Lake and some other key ecological regions [21–23], except Southwest China which is abundant in biodiversity and ecosystem services.

The composition and configuration of different ecosystems within a changing landscape are so influential to the alteration of biochemical and biophysical conditions, that the provision of ecosystem service from these conservation hotspots may be affected [24,25]. In this unique tropical region in China, conservation priorities are rarely related to the management of ecosystem services [26], and the interaction between landscape patterns and ecosystem services is still unclear. For the sub-watershed scale, explorations of the relationship between landscape pattern and ecosystem service has practical implications. This helps us understand the interaction of *LUC* and *BESC* as well as makes understanding ecosystem service variations detected in the local area more intuitive.

Xishuangbanna prefecture, as a typical rainforest area in Southwest China, has been affected by dramatic *LUC* in recent years and is facing enormous threats from human activities [27]. Economic growth principally related to local road construction and urban sprawl has resulted in the destruction of natural forests. *LUC* in Xishuangbanna is characterized by the destruction of the natural forest, the extension of artificial forests such as rubber and tea plantations, and construction areas [28,29]. Furthermore, deforestation here has caused ecological problems as well as the eco-environmental imbalance influenced by *LUC* [30]. Obviously, its residential or construction areas continue increasing at the expense of natural forestland [31–33]. Many local species are threatened by the loss of habitat, such as Asian elephant (*Elephas maximus*), whose endangered status has been blamed on the natural forest shrinking due to farmland cultivation, infrastructure construction and city expansion [34,35]. So, it is crucial to identify the priorities for restoration and provide useful information for future nature conservation and planning in Xishuangbanna.

The goals of this paper are to: (1) quantify *BES*s from 1976, 1990, and 2010 based on land-use dynamics; (2) identify and analyze conservation hotspots with high *BES* values for different years in Xishuangbanna; and (3) assess the impacts of *LUC* on multiple ecosystem services at the scale of both the study area and the sub-watershed. In this study, the InVEST model was applied to calculate and visualize the quantitative changes in *BES*. Conservation priority analysis allows the identification of hotspot areas with high *BES* values within a landscape.

## 2 Materials and methods

### 2.1 Study area

**Fig 1:**
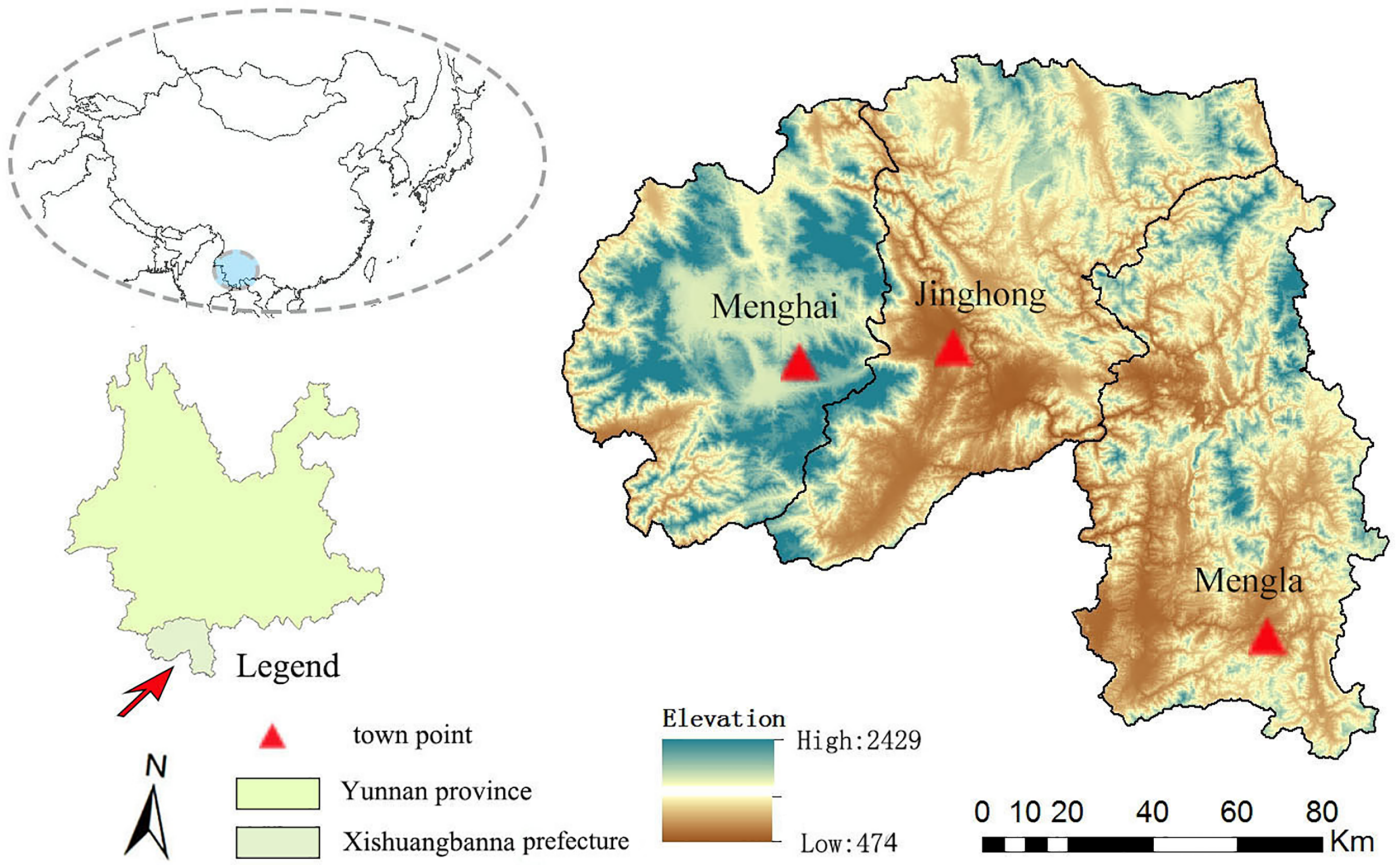
Location of the study area.

Xishuangbanna (21°09′-22°36′N, 99°58′-101°50′E) is located at the very south of the Yunnan province, belonging to a transitional zone from tropical Southeast Asia to subtropical East Asia, which has rich biological diversity and typical rainforest ecosystem [36]. It borders Laos and Myanmar, and consists of Jinghong, Menghai, and Mengla Counties (Fig 1). The total area is about 19120 km^2^, and the elevation varies from 474 m to 2429 m above sea level. The western and eastern regions are areas of higher elevation, while the central region is lower and extensively disturbed by anthropogenic effects. The region’s annual mean temperature is approximately 21°C and its annual precipitation is over l500 mm.

### 2.2 Data sources

Three periods (1976, 1990 and 2010) were chosen for the InVEST model and two periods (1990 and 2010) were used to analyze the relationship between *BES* and landscape pattern. The land-use data were obtained from the Data Centre for Resources and Environmental Sciences, Chinese Academy of Sciences (http://www.resdc.cn/), and validated with an overall accuracy of at least 85% by the ground data. The land uses were classified into eight types: broad-leaved forest, coniferous forest, dry land, paddy field, artificial forest, residential area, shrub grassland and water. A Digital Elevation Model (DEM) data (100m) were provided by the International Scientific & Technical Data Mirror Site, Computer Network Information Centre, Chinese Academy of Sciences (http://www.gscloud.cn/) and were used to generate the sub-watershed map.

All data (or layers) needed in the process of the InVEST modeling are shown in S1 Table. Roads were classified as a national expressway, provincial road, county road, or rural road in our study. The resolution of all data layers was resampled as 100 m×100 m. Buffer distances around residential area and cities were set as 3 km and the relative sensitivity of each habitat type to each threat was identified. In addition, the data on water yield and the value of “carbon pools”, varying from one land use to another, were collected (S1 Table).

### 2.3 InVEST model

The InVEST model was used to calculate habitat quality, water yield and carbon storage in our study. Habitat quality is viewed as a proxy for biodiversity in the InVEST model (Significantly, "habitat quality" is not the same as "biodiversity", as it doesn't include species data) and is estimated by analyzing land cover in conjunction with related threats [9]. There are many rare species that depend heavily on forest and water resources in the tropical region. These different habitat types are treated equally and inputs are assumed to not focus on any particular species, but rather apply to general biodiversity in this model [9]. The formulas are as follows [9]:

$$Q_{xj}=H_{j}(1-(\frac{D_{xj}^{z}}{D_{xj}^{z}+k^{z}}))$$ (1)

$$D_{xj}=\sum_{r=1}^{R}\sum_{y=1}^{Y_{r}}\left(\frac{\omega_{x}}{\sum_{r=1}^{R}\omega_{x}}\right)r_{y}i_{rxy}\beta_{x}S_{jr}$$ (2)

$$i_{rxy}=exp(-(\frac{2.99}{d_{rmax}})d_{xy})$$ (3)

where, *D*_*xj*_ is the total threat level on pixel *x* for *LU*_*j*_; *r* presents the threat layer; *y* indices all grid cells on *r*’s raster map; *Y*_*r*_ indicates the set of grid cells on *r*’s raster map; *d*_*xy*_ is the linear distance between grid cells *x* and *y*; *d*_*rmax*_ is the maximum effective distance of threat *r*’s reach across space; *Q*_*xj*_ means the habitat quality of pixel *x* in *LU*_*j*_; *H*_*j*_ indicate the habitat suitability of *LU* type *j*. Constant *k* is the half-saturation constant and *z* = 2.5 is set as programmed. The final habitat quality values range from 0 to 1. The impact of threats on habitat is mediated by four factors: 1) *ω*_*r*_ indicates the relative impact of each threat (value from 0 to 1); 2) *i*_*rxy*_ indicates the distance between habitat and the threat source and the impact of the threat across space; 3) *β*_*x*_ is the factor that may mitigate the impact of threats on habitat through various protection polices (here *β*_*x*_ = 1); and 4) *S*_*jr*_ ∈ [0, 1] indicates the sensitivity of *LU*_*j*_ to threat *r* where values closer to 1 indicate greater sensitivity, (if *S*_*jr*_ = 0 then *D*_*xj*_ is not a function of threat *r*).

The amount of water for each pixel was calculated based on the Budyko curve method and precipitation data at the sub-watershed scale in the model, and formulas of water yield are as follows [9,37]:

$$Y(x)=(1-\frac{AET(x)}{P(x)})\times P(x)$$ (4)

AET(x)P(x)=1+Kc(lx)ET0(x)P(x)-[1+(Kc(lx)ET0(x)P(x))ω]1ω (5)

$$\omega (x)=Z\frac{AWC(x)}{P(x)}+1.25$$ (6)

where, *Y*(*x*) is the annual water yield for pixel*x*; *AET*(*x*) represents the annual actual evapotranspiration; *P*(*x*) is the annual precipitation; *k*_*c*_(*l*_*x*_) is the vegetation evapotranspiration coefficient associated with the landuse *l*_*x*_; *ET*_0_(*x*) is the reference evapotranspiration of pixel *x*; ω(*x*) is a non-physical parameter to characterize the natural climatic-soil; *AWC*(*x*) is the volumetric plant available water content (mm); and *Z* parameter is a seasonality coefficient corresponding to the seasonal precipitation distribution.

Four carbon “pools” in terrestrial ecosystems (aboveground biomass, belowground biomass, soil, and dead organic matter) are included in the InVEST Carbon Storage and Sequestration model that is used to calculate the carbon storage of study area [9].

$$C_{t}=C_{above}+C_{below}+C_{dead}+C_{soil}$$ (7)

where, *C*_*t*_ is the total carbon density (Mg/ha) for each pixel; *C*_*above*_, *C*_*below*_, *C*_*dead*_ and *C*_*soil*_ are the carbon density (Mg/ha) of aboveground biomass, belowground biomass, dead organic matter, and soil, respectively.

### 2.4 Hotspot recognition and Kernel Density analysis

Hotspots were defined first before overlapping and analyzing *BES*s. Usually, the percentage of top *BES* values for a given area can be used to recognize the hotspots [38]. Given this, we abstracted the top 20% of each layer and overlaid them to gain the final hotspots, which further need to be clipped with the land use map to figure out which kinds of land-use have high ecosystem values in our study area. The Kernel Density of conservation hotspots was also recognized in ArcGIS 10.0 (search radius was set as 2500 m) to explore the general distribution characteristics of these hotspots.

### 2.5 Relationship between BES and landscape pattern

The temporal changes of *BES* were quantified as follows [3]:

$${BESCI}_{x}=\frac{{BES}_{{CUR}_{xj}}-{BES}_{{HIS}_{xi}}}{{BES}_{{HIS}_{xi}}}$$ (8)

$$BESSI=\frac{\sum B{ESCI}_{x}}{n}$$ (9)

$${BESSI}_{mean}=\frac{\sum_{p}^{m}BESSI}{m}$$ (10)

where, *BESCI*_*x*_ is the relative change index of each service *x*, and *BESSI*_*x*_ is the total change of all the considered *BES*, both ranging from -1 to 1. ${BES}_{{CUR}_{xj}}$ and ${BES}_{{HIS}_{xi}}$ are the current and historical states, respectively, of service *x* at times *j* and *i* (here, *j* = 2010, *i* = 1990). *BESSI*>0 means the improvement of local ecological supporting system while *BESSI*<0 indicates the degradation of local ecosystem state. *BESSI*_mean_ is the average change value of *BES* of each sub-watershed; and *m* is the total number of pixels in a sub-watershed.

The landscape pattern indices—Patch Cohesion Index (*COHESION*), Landscape Shape Index (*LSI*), Number of Patches (*NP*), Perimeter-Area Fractal Dimension (*PAFRAC*), and Shannon’s Diversity Index (*SHDI*)—were chosen to represent the landscape pattern of the study area, and were calculated by the method of moving window (2 km) in Fragstats 4.2. Besides the *COHESION*, all of the other indices belong to the negative landscape indices which will increase in value if the degree of landscape integrity declines (or landscape fragmentation increases). The sub-watershed layer was generated by the use of ArcSWAT and resulted from DEM data that is available from NASA online. These catchments were classified into two classes (the negative group and the positive group) based on the average value of *BESSI*_*mean*_. Then, these two groups of catchments were taken as the statistical unit of aforementioned landscape pattern indices to explore the relationship between *BES* and landscape pattern, and to evaluate the hotspot change in terms of the landscape characteristics.

## 3 Results

### 3.1 Changes of BES and land use from 1976 to 2010

**Fig 2:**
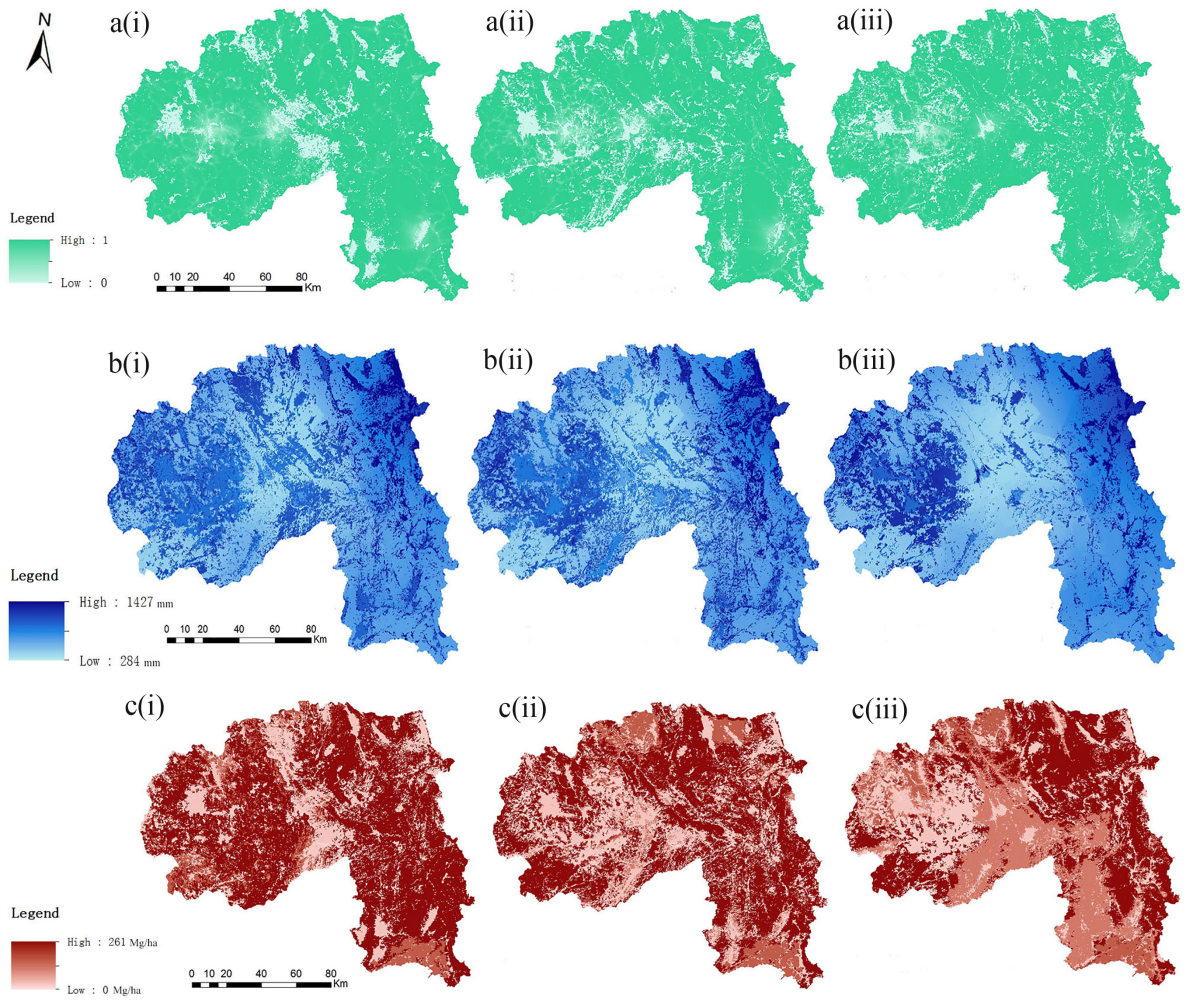
Temporal variations of *BES* ((a), (b), (c) are changes in habitat quality, water yield, and carbon storage respectively; (i), (ii), (iii) represents the years 1976, 1990 and 2010).

The artificial forest area in Xishuangbanna increased from 23.84×10^7^ m^2^ to 492.41×10^7^ m^2^, while the broad-leaved forest area decreased greatly by 31.4% from 1976 to 2010 (S2 Table). Most of the broad-leaved forests were converted to shrub grassland, artificial forest, and paddy field. Another two distinct changes of land use were the continuous growth of residential areas and paddy fields, and the declining trend of dry land. Spatially, land uses in the middle and west regions varied much more intensely than those in the east region where natural forest was dominant (S1 Fig). Changes in the distribution and quantity of three ecosystem services were also obvious (Fig 2). For habitat quality, Menghai County and Mengla County were much better than Jinghong County. Water supply service ranged from 284 mm to 1427 mm. Water supply values in the eastern and western parts were higher than those in the central part of the study area. Carbon storage was always high in the eastern region but reduced in the western areas gradually.

According to the land-use maps, high *BES*s were mostly concentrated on forest land and agro-forest land, suggesting that these land uses are critical for provisioning of the ecosystem services considered here. Comparatively, values of these services were lower in residential areas, especially in the central part of the region. Ecosystem service quantities in 1976 were much higher than the ones in 1990or 2010. As a whole, the region experienced a greater loss in *BES*s during the study period.

**Fig 3:**
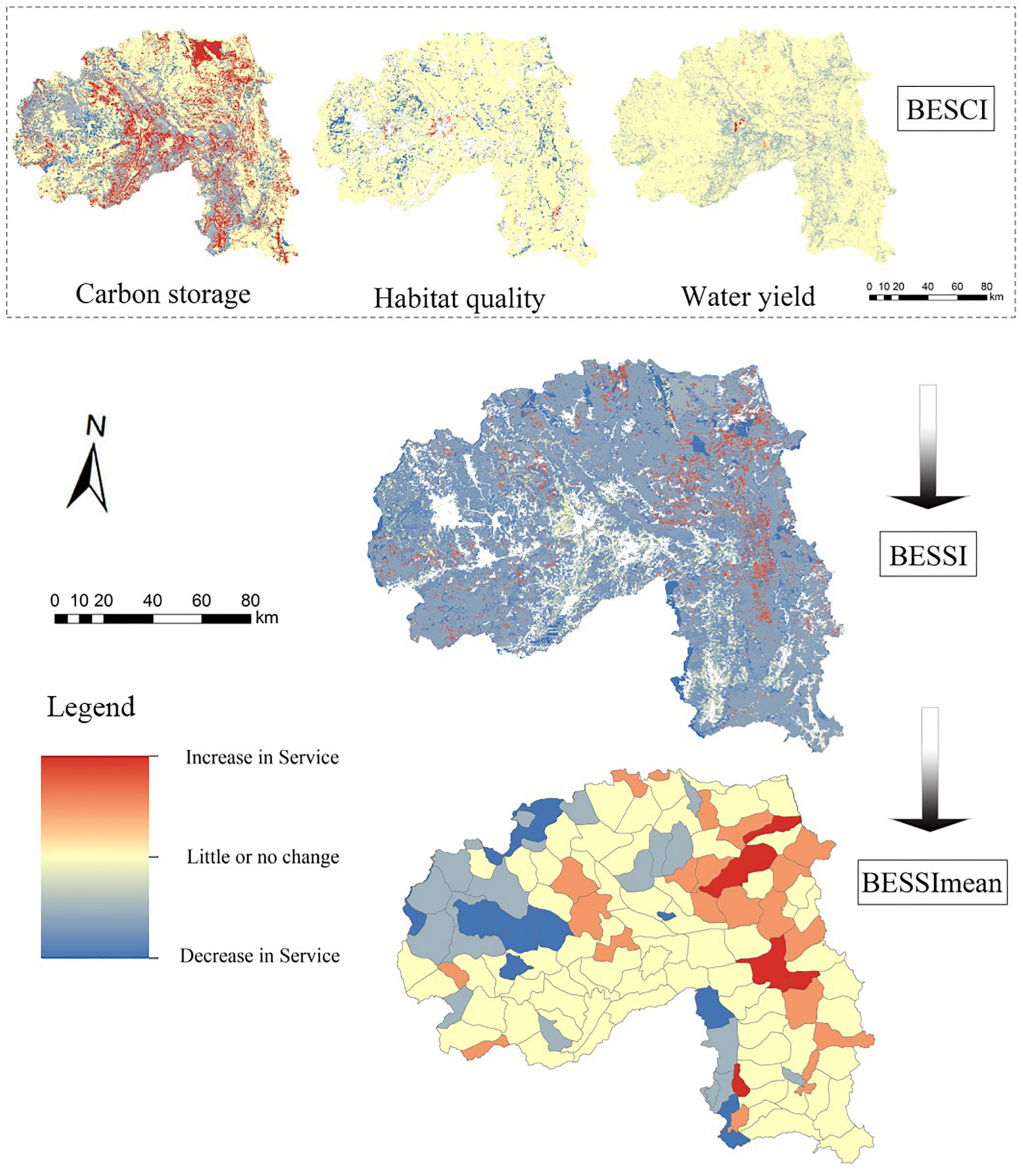
Changes of *BES* between 1990 and 2010.

The *BESCI* index clearly showed the spatial variations of different services. Except for the carbon storage, the other two services increased little in quantity from 1990 to 2010 (Fig 3). The *BESSI* value ranged from -0.44 to 0.77, from which we found that only a few areas obviously increased in *BES* and most of them were distributed in the eastern region. There were 138 sub-watersheds distributed in Xishuangbanna and twenty-five percent of the sub-basins belonged to the negative group. However, most of the sub-watersheds remained relatively stable in *BES*s during this period.

### 3.2 Hotspot identification

**Fig 4:**
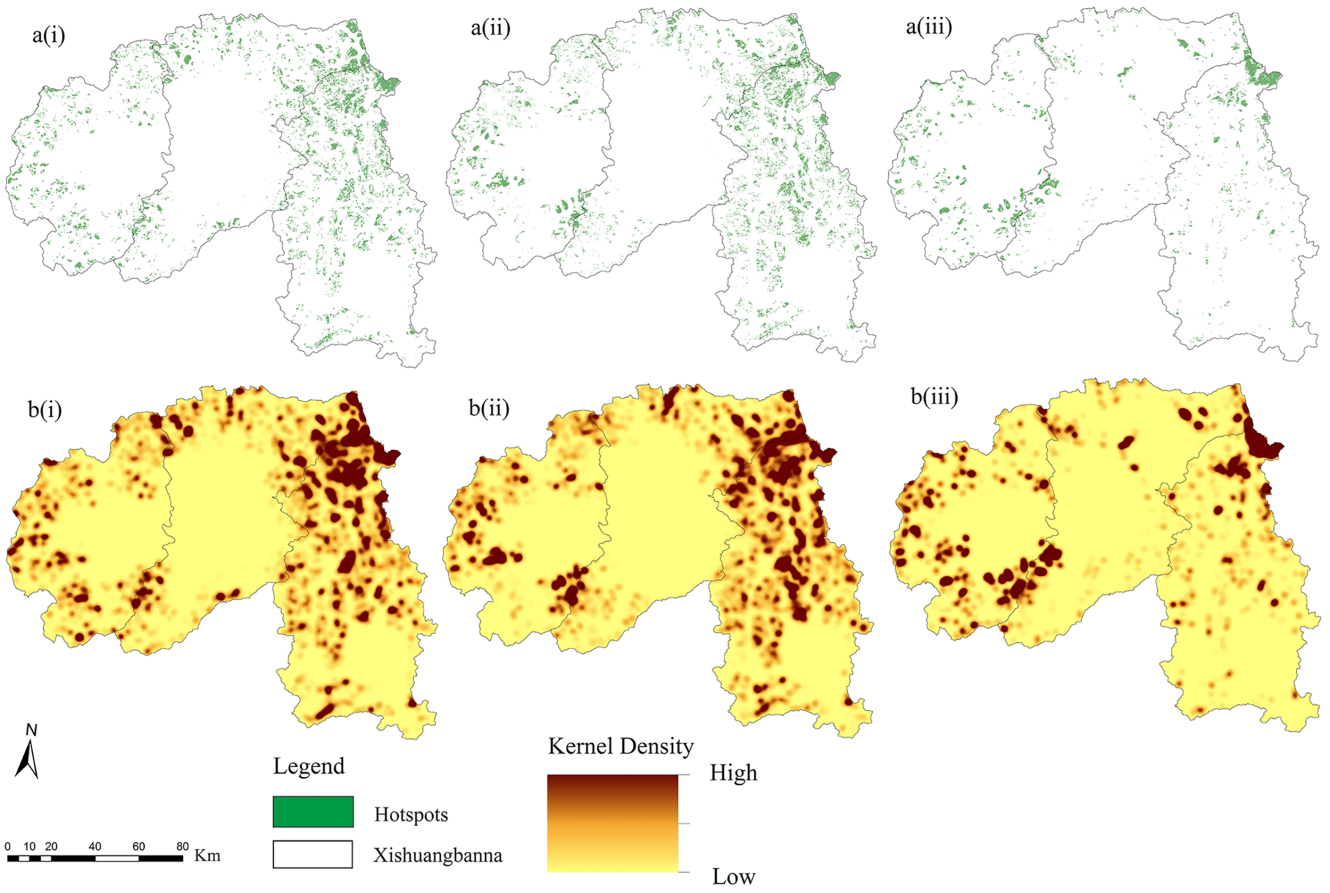
Distributions of hotspots during the study periods ((a), (b) are the distribution and the Kernel Density of hotspots, respectively; (i), (ii), (iii) represent the year of 1976, 1990 and 2010).

Assessment of the cumulative status of *BES*s can generate a picture quite different from individual ecosystem service evaluation. The hotspots were recognized by overlaying biodiversity and the two basic ecosystem services, and further clipped by the corresponding land-use layers. Distributions of hotspots tended to be more scattered and the quantity of hotspots decreased greatly from 1.25×10^5^ ha to 0.63×10^5^ ha, in total reduced by nearly 50% during thirty-five years (Table 1). Few hotspots were identified around Jinghong County, while most hotspots were located in the administrative region of Mengla County. However, the hotspot densities also declined in the eastern region, and the high-density areas significantly decreased, especially in 2010 (Fig 4).

**Table 1:** Statistics of the land-use of hotspots.

Land cover	Area (ha)		
	2010	1990	1976
Broad-leaved forest	11000.47	36831.32	52617.01
Coniferous forest	377.78	4371.02	2781.97
Dry land	353.31	1005.70	385.61
Paddy field	1268.52	631.16	205.79
Artificial forest	814.14	729.12	88.25
Residential area	0	0	0
Shrub grassland	49677.94	73446.11	68805.86
Water	18.61	25.95	11.67
Total area	63510.83	117040.39	124896.17

## 4 Discussion

### 4.1 LU impacts on BES and hotspots

The habitat quality increased in the middle of the study area because of the extension of artificial forest which was also treated as a habitat in our study. Although artificial afforestation could have negative effects on forest biodiversity and natural connectivity compared with natural forests [39], the main purpose and function of plantation in deforested regions could contribute to land recovery and biodiversity protection to reduce ecological degradations to some extent [40,41]. As for Jinghong County, rubber was the main type of plantation and became the selective habitat of Asian elephant and intermediate habitat of some amphibians [42,43]. Because of the field-data limitation, only habitat quality from1990 and 2010 were validated by animal occurrence records from previous studies [44,45]. Based on the data of local animal monitoring points, about 35% Asian elephant and 70% green peafowl occurred in the top 20% of habitat quality in 1990. For Asian elephant, this proportion was up to 42% in 2010 (S2 Fig). The main reason why the proportion of Asian elephants in the top 20% was less than 50% might be attributed to the fact that elephants may usually show up in dry land or residential areas which are not their suitable habitats [43]. Regardless, the results of *BES* for habitat quality can indicate the biodiversity levels in Xishuangbanna for certain species.

According to our results, high values of water supply occurred in the areas that were high in altitude and covered by shrub grassland, paddy fields or natural forests. The central part of the study area was low in water yield, because evapotranspiration is relatively high with decreasing vegetation coverage and increasing constructed areas. The annual precipitation change was not detectable for the whole region, but the changes in evapotranspiration are obvious because of the change of surface structures. Impervious surfaces, the typical land type affected by human activities, have relatively less inability to store water, but have a strong thermal storage capacity [46]. In contrast, approximately two-thirds of the total water yield was produced by grasslands and forest ecosystems. Referring to some previous studies [24,47], the results of our study also showed that abundant shrub or grass planting in the catchment can increase water yield and have positive quantified effects on the hydrological cycle. Besides these habitat types, the tropical rain forest also enhances the landscape resilience to hydrological change, and plays a significant role in water conservation [48,49].

Information about how much and where carbon is stored is vital to landscape management, and can be used by the government to decide the main use (for protection, harvest, or development) of targeted sites. Carbon storage in 1976 was much higher than in the other two periods, especially in the western and eastern regions covered by large areas of natural forest. However, storage quantities of carbon in the western areas, where broad-leaved forest was converted into shrub grassland or paddy fields, gradually declined during the study period. The biomass of artificial forest was much lower than that of local tropical natural forest, as well as the shrub grassland [50,51]. According to the *BESCI* index of carbon storage (Fig 3), we can detect the obvious increase in carbon stock which was caused by the plantation expansion in 2010.

Compared with other land cover types, broad-leaved forest and shrub grassland occupied the largest proportion of the hotspots. The percentage of broad-leaved forest decreased from 42.13% to 17.32% during thirty-five years; while the proportion of shrub grassland increased gradually, from 55.09% to 78.22% (Table 2). Because of the high altitude and surrounding paddy fields which increased the humidity of the surrounding air and reduced the value of *ET*_*0*_, the shrub grassland had high values and occupied an increasing proportion of hotspots in terms of the service of water yield. According to the definition, hotspots were the areas that belonged to the top 20% of each layer simultaneously. However, because the top 20% water yield had the smallest area percentage compared with the other services, the water yield service was found to be the limiting factor of hotspot identification in our study (Table 2). Moreover, the high standard deviation value of water yield showed that it had a high variability and spread out over a wider range. Surface differences between the residential areas and the broad-leaved forest are significant, leading to an obvious difference in water yield.

**Table 2:** Area proportions of *BES*s ranking for hotspots.

*BES*	Ranking	1990	2010
Habitat quality	20%	81.06%	84.01%
	20–40%	4.56%	3.73%
	40%-100%	14.38%	12.25%
	Standard Deviation	0.35	0.33
Water yield	20%	20.27%	19.36%
	20–40%	18.32%	20.25%
	40%-100%	61.40%	60.40%
	Standard Deviation	217.26	191.84
Carbon storage	20%	81.55%	61.60%
	20–40%	4.57%	26.37%
	40%-100%	13.89%	12.04%
	Standard Deviation	105.53	94.88

Further analyses that focused at the scale of sub-watersheds would be more meaningful to informing practice (like local landscape management, land-use planning, resource conservation and so on). According to our study results, from both 1990 and 2010, hotspots that were located in the sub-watershed whose *BESSI*>0 were more than three times the ones located in the sub-watershed whose *BESSI*<0. That is to say, most of hotspots were distributed in the areas with increased *BES*, suggesting that these sub-basins played a key role in safeguarding regional ecosystem services. Then, what about different kinds of sub-watersheds featured in the landscape characteristics that can describe the landscape pattern? We discovered that all the negative landscape indices (*LSI*, *NP*, *PAFRAC*, and *SHDI*) increased as *BESSI*<0, indicating that affected sub-watersheds would suffer from landscape fragmentation and ecosystem services loss at the same time (Table 3).

**Table 3:** Changes of landscape pattern indices based on sub-watershed groups.

Sub-watersheds	Landscape pattern index	1990	2010	The absolute change rate (%)	The relative change rate (%)
*BESSI*_*mean*_<0	COHESION	95.44	94.56	-0.88	-0.92
	LSI	2.41	2.62	0.21	8.74
	NP	8.98	11.25	2.27	25.32
	PAFRAC	1.35	1.36	0.01	0.52
	SHDI	0.71	0.84	0.13	18.37
*BESSI*_*mean*_>0	COHESION	94.91	95.84	0.93	0.98
	LSI	2.56	2.2	-0.36	-14.02
	NP	9.99	8.65	-1.35	-13.47
	PAFRAC	1.37	1.34	-0.03	-1.91
	SHDI	0.77	0.63	-0.15	-18.97

### 4.2 Attention to certain hotspots

The National Nature Reserve in Xishuangbanna includes five national sub-reserves: Mangao (7870 ha), Mengyang (99840 ha), Menglun (10933 ha), Mengla (926833 ha), and Shangyong (31184 ha). However, all of these sub-reserves occupied only 15%–30% of the total hotspot areas during the thirty-five years. There are at least two main reasons for this result. On the one hand, contributions of five nature sub-reserves that were formally established in 1981, were not only based on the biodiversity state, but also in the light of related rules according to Regulations of the People's Republic of China on Nature Reserves (http://www.gov.cn/flfg/2005-09/27/content_70636.htm). These reserves do not fully consider ecosystem services, since the *BES* theory had not been proposed until the 21st century [52,53] and the applications of *BES* came even later in China. On the other hand, the locations of reserves depend largely on actual situations, such as the growth of vegetation, animal distributions, social conditions, topography and so on. It is undeniable that all of these factors could not be adequately considered in one model. Due to these aspects, the deviations that occurred between hotspots and nature reserves cannot be ignored.

It is true that ecosystem service degradation can weaken the environment’s ability to support the diversity of livelihoods [54]. For the areas with high *BES* values, hotspots, especially those outside the nature sub-reserves, should be protected in advance and viewed as potential areas for further conservation. What’s more, about twenty-three percent of the hotspots are associated with the negative group (*BESSI*_*mean*_<0). These hotspots are also sensitive to landscape fragmentation caused by human land use practices, and therefore need extra protection. So, land managers should pay much attention to these key areas. As a whole, in the eastern region in the Xishuangbanna prefecture, more conservation areas should be designated.

### 4.3 Limitations of BES in the study region

This study explored local ecological hotspots with limited ecosystem services as well as their temporal change. Habitat quality, water yield, and carbon storage were selected in our study, but there is a highly likelihood of deriving different results if different arrays of ecosystem services were chosen. Nevertheless, data deficiency prevented us from modeling all regional related ecosystem services available in the InVEST model. In addition, limitations of the InVEST model—its simplified modeling processes and inability to account for seasonal variability—cannot be ignored [55]. For example, the water module is unable to cover the full hydrologic cycle and the biodiversity index only focuses on habitat quality without considering species richness or other relevant indices of biodiversity [9]. Users should be aware of the uncertainties represented using this model and strengthen service simulations by field validation.

Hotspots mapped by overlaying different layers have represented key protected regions of biodiversity as well as other key ecosystem services in human dominated landscapes. What’s more, all the hotspots and areas with high values in each service have far-ranging policy implications in maintaining a sustainable eco-environment.

## 5 Conclusions

In Xishuangbanna, land cover changed significantly from 1976 to 2010 with the sharp decline of broad-leaved forest and the continuous increase of paddy fields, artificial forest and shrub grassland. Land-use type and structure dramatically affected *BES* in different ways. Total values of ecosystem services (including habitat quality, carbon storage and water yield) decreased with time. Correspondingly, the hotspots with high *BES* values shrunk and their distributions tended to be more scattered. At the sub-watershed scale, though most areas showed a stable trend in services, the values of *BES* were generally high in eastern sub-watersheds compared with western ones. However, sub-watersheds that decreased in services included nearly a quarter of the hotspots, which were more sensitive to landscape fragmentation. The prioritized hotspot areas which were composed primarily of broad-leaved forest and shrub grassland were regarded as potential conservation areas in addition to the current sub-reserves. These hotspots can provide multiple ecosystem services that are beneficial not only to local species, but also to landscape sustainability.

Our methods make full use of readily available data; therefore, it is applicable to other areas or higher level regions for hotspots identification. A better understanding of land-use effects on biodiversity conservation is significant for ensuring sustainable growth of environmental protection and social development. In conclusion, this study provides sound information to locate Xishuangbanna hotspots and sensitive sub-watersheds, which is helpful to minimize the degradation of ecosystem services in future environmental conservation.

## Supporting information

S1 Table Data requirements for the three sub-models. (DOCX)

S2 Table The transfer matrix of land use areas from 1976 to 2010 (m^2^). (DOCX)

S1 Fig Land-use of 1976, 1990, and 2010. (TIF)

S2 Fig Validation of the habitat quality. (TIF)

## References

[1] Faith DP, Magallon S, Hendry AP, Conti E, Yahara T, Donoghue MJ. Evosystem services: an evolutionary perspective on the links between biodiversity and human well-being. Current Opinion in Environmental Sustainability. 2010; 2(1–2): 66–74.

[2] Gardner TA, Barlow J, Sodhi NS, Peres CA. A multi-region assessment of tropical forest biodiversity in a human-modified world. Biological Conservation. 2010; 143(10): 2293–2300.

[3] Leh MDK., Matlock MD, Cummings EC, Nalley LL. Quantifying and mapping multiple ecosystem services change in West Africa. Agriculture Ecosystems & Environment. 2013; 165: 6–18.

[4] Margules CR, Pressey RL. Systematic conservation planning. Nature. 2000; 405(6783): 243–253. doi: 10.1038/35012251 10821285 10.1038/35012251

[5] van Jaarsveld AS, Freitag S, Chown SL, Muller C, Koch S, Hull H, et al Biodiversity assessment and conservation strategies. Science. 1998; 279(5359): 2106–2108. 9516111 10.1126/science.279.5359.2106

[6] Nackoney J, Williams D. Conservation prioritization and planning with limited wildlife data in a Congo Basin forest landscape: assessing human threats and vulnerability to land use change. Journal of Conservation Planning. 2012; 8: 25–44.

[7] Mace GM, Norris K, Fitter AH. Biodiversity and ecosystem services: a multilayered relationship. Trends in Ecology & Evolution. 2012; 27(1): 19–26. 21943703 10.1016/j.tree.2011.08.006

[8] Polasky S, Nelson E, Pennington D, Johnson KA. The impact of land-use change on ecosystem services, biodiversity and returns to landowners: A case study in the state of Minnesota. Environmental & Resource Economics. 2011; 48(2): 219–242.

[9] Sharp R, Tallis HT, Ricketts T, Guerry AD, Wood SA., Chaplin-Kramer R, et al InVEST 3.1.1 User's Guide. The Natural Capital Project, Stanford 2014.

[10] Burkhard B, Kroll F, Nedkov S, Muller F. Mapping ecosystem service supply, demand and budgets. Ecological Indicators. 2012; 21: 17–29.

[11] Burkhard B, Petrosillo I, Costanza R. Ecosystem services—Bridging ecology, economy and social sciences. Ecological Complexity. 2010; 7(3): 257–259.

[12] Daily GC, Polasky S, Goldstein J, Kareiva PM, Mooney HA, Pejchar L, et al Ecosystem services in decision making: time to deliver. Frontiers in Ecology and the Environment. 2009; 7(1): 21–28.

[13] Egoh B, Reyers B, Rouget M, Bode M, Richardson DM. Spatial congruence between biodiversity and ecosystem services in South Africa. Biological Conservation. 2009; 142(3): 553–562.

[14] Egoh B, Rouget M, Reyers B, Knight AT, Cowling RM, van Jaarsveld AS, et al Integrating ecosystem services into conservation assessments: A review. Ecological Economics. 2007; 63(4): 714–721.

[15] Fu BJ, Zhang L. Land-use change and ecosystem services: concepts, methods and progress. Progress in Geography.2014; 33(4): 441–446.

[16] Fu BJ, Wang S, Su CH, Forsius M. Linking ecosystem processes and ecosystem services. Current Opinion in Environmental Sustainability. 2013; 5(1): 4–10.

[17] Millennium Ecosystem Assessment. Ecosystems and human well-being synthesis: a report of the Millennium Ecosystem Assessment. Washington DC: Island Press; 2005.

[18] Nelson E, Sander H, Hawthorne P, Conte M, Ennaanay D, Wolny S, et al Projecting global land-use change and its effect on ecosystem service provision and biodiversity with simple models. Plos One 2010; 5(12): e14327–e14327. doi: 10.1371/journal.pone.0014327 21179509 10.1371/journal.pone.0014327 PMC3002265

[19] Sanchez-Canales M, Lopez Benito A, Passuello A, Terrado M, Ziv G, Acuna V, et al Sensitivity analysis of ecosystem service valuation in a Mediterranean watershed. Science of the Total Environment. 2012; 440: 140–153. doi: 10.1016/j.scitotenv.2012.07.071 22925484 10.1016/j.scitotenv.2012.07.071

[20] Nelson E, Mendoza G, Regetz J, Polasky S, Tallis H, Cameron DR, et al Modeling multiple ecosystem services, biodiversity conservation, commodity production, and tradeoffs at landscape scales. Frontiers in Ecology and the Environment. 2009; 7(1): 4–11.

[21] Li XW, Li MD, Dong SK, Shi JB. Temporal-spatial changes in ecosystem services and implications for the conservation of alpine rangelands on the Qinghai-Tibetan Plateau. Rangeland Journal. 2015; 37(1): 31–43.

[22] Pan T, Wu S, Dai E, Liu Y. Spatiotemporal variation of water source supply service in Three Rivers Source Area of China based in InVEST model. Chinese Journal of Applied Ecology. 2013; 24(1): 183–189. 23718008

[23] Sun C, Zhen L, Wang C, Hu J, Du B. Biodiversity simulation of Poyang Lake wetlands by InVEST model under different scenarios. Resources and Environment in the Yangtze Basin. 2015; 24(7): 1119–1125.

[24] Deng XZ, Li ZH, Huang JK, Shi QL, Li YF, Zhang RR, et al Reviews on impact assessments of land-use change on key ecosystem services In: Zhan J, editor. Impacts of Land-use Change on Ecosystem Services. Series: Springer Geography; 2015 pp. 1–35.

[25] Tardieu L, Roussel S, Thompson JD, Labarraque D, Salles JM. Combining direct and indirect impacts to assess ecosystem service loss due to infrastructure construction. Journal of Environmental Management. 2015; 152: 145–157. doi: 10.1016/j.jenvman.2015.01.034 25621389 10.1016/j.jenvman.2015.01.034

[26] Grêt-Regamey A, Rabe SE, Crespo R, Lautenbach S, Ryffel A, Schlup B. On the importance of non-linear relationships between landscape patterns and the sustainable provision of ecosystem services. Landscape Ecology. 2014; 29: 201–212.

[27] Laurance WF. Have we overstated the tropical biodiversity crisis? Trends in Ecology & Evolution. 2007; 22(2): 65–70. 17011069 10.1016/j.tree.2006.09.014

[28] Li H, Ma Y, Guo Z, Liu W. Land use/land cover dynamic change in Xishuangbanna based on RS and GIS technology. Journal of Mountain Science. 2007; 25(3): 280–289.

[29] Zhang PF, Xu JC, Wang MX, Deng XQ. Spatial and temporal dynamics of rubber plantation and its impacts on tropical forest in Xishuangbanna. Remote Sensing for Land &Resources. 2006; 3(69): 51–55.

[30] Zhang J, Cao M. Tropical forest vegetation of Xishuangbanna, SW China and its secondary changes, with special reference to some problems in local nature conservation. Biological Conservation. 1995; 73: 229–238.

[31] He CG, Feng Y, Yang YP. Research on evolution process and driving factors of forest landscape in Xishuangbanna. Yunnan Geographic Environment Research. 2008; 20(5): 12–17.

[32] Liu X, Feng Z, Jiang L, Zhang J. Spatial-temporal pattern analysis of land use and land cover change in Xishuangbanna. Resources Science. 2014; 36(2): 233–244.

[33] Zhu H, Xu ZF, Wang H, Li BG. Over 30-year changes of floristic composition and population structure from an Isolated fragment of tropical rain froest in Xishuangbanna. Acta Botanica Yunnanica. 2001; 23(4): 415–427.

[34] Zhang L, Wang N, Wang YN, Ma LC. A preliminary study on the habitat and behaviors of Asian Elephant (Elephas maximus) in Simao, Yunnan, China. Acta Theriologica Sinica. 2003; 23(3): 185–192.

[35] Zomer RJ, Trabucco A, Wang MC, Lang R, Chen HF, Metzger MJ, et al Environmental stratification to model climate change impacts on biodiversity and rubber production in Xishuangbanna, Yunnan, China. Biological Conservation. 2014; 170: 264–273.

[36] Zhu H. Forest vegetation of Xishuangbannan, south China., Forest Studies in China. 2006; 8(2): 1–58.

[37] Budyko MI. Climate and life. Orlando: Academic Press; 1974.

[38] Anderson BJ, Armsworth PR, Eigenbrod F, Thomas CD, Gillings S, Heinemeyer A, et al Spatial covariance between biodiversity and other ecosystem service priorities. Journal of Applied Ecology. 2009; 46(4): 888–896.

[39] Rojas C, Pino J, Basnou C, Vivanco M. Assessing land-use and -cover changes in relation to geographic factors and urban planning in the metropolitan area of Concepcion (Chile). Implications for biodiversity conservation. Applied Geography. 2013; 39: 93–103.

[40] Brook BW, Sodhi NS, Ng PKL. Catastrophic extinctions follow deforestation in Singapore. Nature. 2003; 424(6947): 420–423. doi: 10.1038/nature01795 12879068 10.1038/nature01795

[41] Meng Q. Discussion on effects of plantation on biodiversity. World forestry research. 2006; 19(5): 1–6.

[42] Behm JE, Yang XD, Chen J. Slipping through the cracks: rubber plantation is unsuitable breeding habitat for frogs in Xishuangbanna, China. Plos One. 2013; 8(9): 274–274. 10.1371/journal.pone.0073688 PMC3769397 24040026

[43] Lin L, Jin YF, Yang HP, Luo AD, Guo XM, Wang LF, et al Evaluation of habitat for Asian elephants (Elephas maximus) in Xishuangbanna, Yunnan, China. Acta Theriologica Sinica. 2015; 35(1).

[44] He QC, Wu ZL, Xu FG, Guo XM, Zhu ZY. Human-elephant relations becoming crisis in Xishuangbanna, Southwest China In: Hong SK, Bogaert J, Min Q, editors. Biocultural Landscapes: Diversity, Functions and Values. Dordrecht: Springer Netherlands; 2014 pp. 69–80.

[45] Luo AD, Dong YH. Investigation on the current status of distribution and population of the green peafowl in Xishuangbanna. Chinese Journal of Ecology. 1998; 17(5): 6–10.

[46] Ramamurthy P, Bou-Zeid E. Contribution of impervious surfaces to urban evaporation. Water Resources Research. 2014; 50(4): 2889–2902.

[47] Nosetto MD, Jobbagy EG, Paruelo JM. Land-use change and water losses: the case of grassland afforestation across a soil textural gradient in central Argentina. Global Change Biology. 2005; 11(7): 1101–1117.

[48] Ojea E, Martin-Ortega J. Understanding the economic value of water ecosystem services from tropical forests: A systematic review for South and Central America. Journal of Forest Economics. 2015; 21(2): 97–106.

[49] Portillo-Quintero C, Sanchez-Azofeifa A, Calvo-Alvarado J, Quesada M, Santo MMD. The role of tropical dry forests for biodiversity, carbon and water conservation in the neotropics: lessons learned and opportunities for its sustainable management. Regional Environmental Change. 2015; 15(6): 1039–1049.

[50] Jobbagy EG, Jackson RB. The vertical distribution of soil organic carbon and its relation to climate and vegetation. Ecological Applications. 200; 10(2): 423–436.

[51] Song Q, Zhang Y. Biomass carbon sequestration and its potential of rubber plantation in Xishuangbanna, Southwest China. Chinese Journal of Ecology. 2010; 29(10): 1887–1891.

[52] Balvanera P, Daily GC, Ehrlich PR, Ricketts TH, Bailey SA, Kark S, et al Conserving biodiversity and ecosystem services. Science. 2001; 291(5511): 2047–2047. 11256386 10.1126/science.291.5511.2047

[53] Singh SP. Balancing the approaches of environmental conservation by considering ecosystem services as well as biodiversity. Current Science. 2002; 82(11): 1331–1335.

[54] Smajgl A, Xu J, Egan S, Yi ZF, Ward J, Su Y. Assessing the effectiveness of payments for ecosystem services for diversifying rubber in Yunnan, China. Environmental Modelling & Software. 2015; 69: 187–195.

[55] Vigerstol KL, Aukema JE. A comparison of tools for modeling freshwater ecosystem services. Journal of Environmental Management. 2011; 92(10): 2403–2409. doi: 10.1016/j.jenvman.2011.06.040 21763063 10.1016/j.jenvman.2011.06.040

