# Supplementary material for: Spatio-temporal variations of conservation hotspots based on ecosystem services in Xishuangbanna, Southwest China
Source: PLoS One. 2017 Dec 12;12(12):e0189368. doi: 10.1371/journal.pone.0189368 (PMC5726655; doi:10.1371/journal.pone.0189368)
Supplement: S1 Table — (DOCX) [file pone.0189368.s001.docx]

S1 Table Data requirements for the three sub-models

| Habitat quality | Descriptions and Data sources | Carbon storage | Data sources | Water yield | Data sources |
| --- | --- | --- | --- | --- | --- |
| Land cover map | Land use data | Land cover map | Land use data | Land cover map | Land use data |
| Threat data  - Threat types  - Threat impact distances  - Relative impact weights  - Decay functions for threats  Habitat types  Sensitivity of habitat types to each threat  Threat source maps  Half-saturation constant | Road and residential areas  Based on the study of Liu (Liu et al., 2008)  Comprehensive knowledge and InVEST guides  Broad-leaved forest, coniferous forest, artificial forest and shrub-grass land  Comprehensive knowledge and InVEST samples  Based on the threat data  0.06^[[1]](#footnote-1)^ | Carbon pools  - Carbon in aboveground biomass  - Carbon in belowground biomass  - Carbon in soil  - Carbon in dead organic matter | Referred to IPCC (IPCC, 2006), Lv (Lv et al., 2007), Chen (Chen et al., 2002) ,Song (Song and Zhang, 2010) and InVEST guides | Root restricting layer depth  Mean precipitation  Plant available water content  Average annual reference evapotranspiration  Watersheds  Biophysical table  - Root depth  - *Kc*  Z parameter | Harmonized World Soil Databaseand and field investigations  Chinese National Metrological Information  Referred to soil type and the correspondent data provided by Zhou (Zhou, 2003)  Estimated by the pan evaporation (Allen, 1998) and interpolated in ArcGIS 10.0 by Kriging interpolation.  Resulted from DEM data by the use of ArcSWAT  Based on the land uses (Allen, 1998; Sharp, 2014)  7^[[2]](#footnote-2)^ |

References

Allen R G, Pereira LS, Raes D, Smith M. Crop evapotranspiration. Guidelines for computing crop water requirements, FAO Irrigation and Drainage. Food and Agriculture Organization of the United Nations, Rome, Italy. 1998. pp. 56.

Chen HW, Feng X, Liu YG, Zhou Y, Wang MD, Li LF, Meng M. Study of Biomass of Six Kinds of Plantations in Xishuangbanna. Yunan Forestry Science and Technology. 2002; 3: 19-22.

IPCC Guidelines for National Greenhouse Gas Inventories. 2006.

Liu, SL, Wen MX, Cui BS, Fu W, Yang M. Definition and Spatial Differentiation of Road-effect Zone:A Case Study in Longitudinal Range-Gorge Region. Progress in Geography. 2008; 27(4): 122-128.

Lv X, Tang J, He Y, Duan W, Song J, Xu H, Zhu S. Biomass and its allocation in tropical seasonal rain forest in Xishuangbanna, southwest China. *Acta Phytoecologica Sinica.*2007; 31(1): 11-22.

Sharp R, Tallis HT, Ricketts T, Guerry AD, Wood SA., Chaplin-Kramer R, Nelson E, Ennaanay D, Wolny S, Olwero N, Vigerstol K, Pennington D, Mendoza G, Aukema J, Foster J, Forrest J, Cameron D, Arkema K, Lonsdorf E, Kennedy C, Verutes G, Kim CK, Guannel G, Papenfus M, Toft J, Marsik M, Bernhardt J, Griffin R, Glowinski K, Chaumont N, Perelman A, Lacayo M, Mandle L, Hamel P, Vogl AL. InVEST 3.1.1 User's Guide. The Natural Capital Project, Stanford. 2014.

Song Q, Zhang Y. Biomass carbon sequestration and its potential of rubber plantation in Xishuangbanna, Southwest China. *Chinese Journal of Ecology*. 2010; 29(10): 1887-1891.

Zhou WZ. A Study on Available Water Capacity of Main Soil Types in China Based on Geographic Information System. M. Sc. Thesis, Nanjing Agricultural University. Nanjing. 2003.

1. Half of the highest grid cell degradation value of the study area (Sharp et al. 2014). [↑](#footnote-ref-1)
2. Estimated by the relationship with ω (Sharp et al, 2014). [↑](#footnote-ref-2)
