# Supplementary material for: Spatio-temporal variations of conservation hotspots based on ecosystem services in Xishuangbanna, Southwest China
Source: PLoS One. 2017 Dec 12;12(12):e0189368. doi: 10.1371/journal.pone.0189368 (PMC5726655; doi:10.1371/journal.pone.0189368)
Supplement: S2 Table — (DOCX) [file pone.0189368.s002.docx]

| Land uses in different years | The year behind | | | | | | | |  |
| --- | --- | --- | --- | --- | --- | --- | --- | --- | --- |
|  | Broad-leaved forest  (10^7^) | Coniferous forest  (10^7^) | Dry land  (10^7^) | Paddy field  (10^7^) | Artificial forest  (10^7^) | Residential area  (10^7^) | Shrub-grass land  (10^7^) | Water  (10^7^) | Total area  (10^7^) |
| **1976-1990** |  | | | | | | | | |
| Broad-leaved forest | 753.51 | 65.46 | 75.29 | 48.79 | 48.42 | 0.01 | 206.80 | 1.27 | 1199.54 |
| Coniferous forest | 100.26 | 60.74 | 5.41 | 1.64 | 1.89 | 0.00 | 15.62 | 0.10 | 185.66 |
| Dry land | 38.55 | 7.71 | 24.03 | 14.71 | 9.34 | 0.00 | 22.90 | 0.51 | 117.74 |
| Paddy field | 6.07 | 0.52 | 7.05 | 42.97 | 9.07 | 0.18 | 10.89 | 1.04 | 77.77 |
| Artificial forest | 16.25 | 4.48 | 0.52 | 0.80 | 0.37 | 0 | 1.41 | 0.00 | 23.84 |
| Residential area | 0 | 0 | 0 | 0 | 0 | 0.07 | 0 | 0 | 0.07 |
| Shrub-grass land | 112.72 | 17.43 | 14.46 | 22.95 | 16.47 | 0.10 | 88.49 | 1.02 | 273.64 |
| Water | 0.36 | 0.06 | 0.47 | 1.11 | 0.40 | 0.00 | 0.64 | 1.43 | 4.46 |
| **Total area** | 1027.72 | 156.38 | 127.24 | 132.97 | 85.95 | 0.35 | 346.75 | 5.35 | 1882.72 |
| **1990-2010** |  | | | | | | | | |
| Broad-leaved forest | 647.55 | 37.32 | 29.38 | 56.91 | 255.74 | 0 | 95.38 | 0.25 | 1122.52 |
| Coniferous forest | 52.40 | 91.29 | 0.80 | 3.48 | 4.75 | 0 | 5.31 | 0.02 | 158.06 |
| Dry land | 15.38 | 0.93 | 35.79 | 19.51 | 52.93 | 0.07 | 35.48 | 0.13 | 160.22 |
| Paddy field | 6.29 | 0.88 | 9.63 | 68.03 | 35.16 | 1.52 | 32.72 | 1.95 | 156.18 |
| Artificial forest | 8.00 | 0.41 | 7.68 | 10.32 | 51.92 | 0.04 | 26.23 | 0.05 | 104.64 |
| Residential area | 0 | 0 | 0 | 0.12 | 0 | 0.13 | 0.09 | 0 | 0.35 |
| Shrub-grass land | 93.59 | 3.77 | 18.24 | 43.73 | 89.98 | 0.01 | 123.68 | 0.44 | 373.43 |
| Water | 0.22 | 0.01 | 0.13 | 2.06 | 1.93 | 0.01 | 0.72 | 2.02 | 7.11 |
| **Total area** | 823.44 | 134.60 | 101.64 | 204.17 | 492.41 | 1.80 | 319.60 | 4.87 | 2082.51 |

S2 Table The transfer matrix of land use areas from 1976 to 2010 (m^2^)
