# Supplementary figures and images for: Spatio-temporal variations of conservation hotspots based on ecosystem services in Xishuangbanna, Southwest China
Source: PLoS One. 2017 Dec 12;12(12):e0189368. doi: 10.1371/journal.pone.0189368 (PMC5726655; doi:10.1371/journal.pone.0189368)

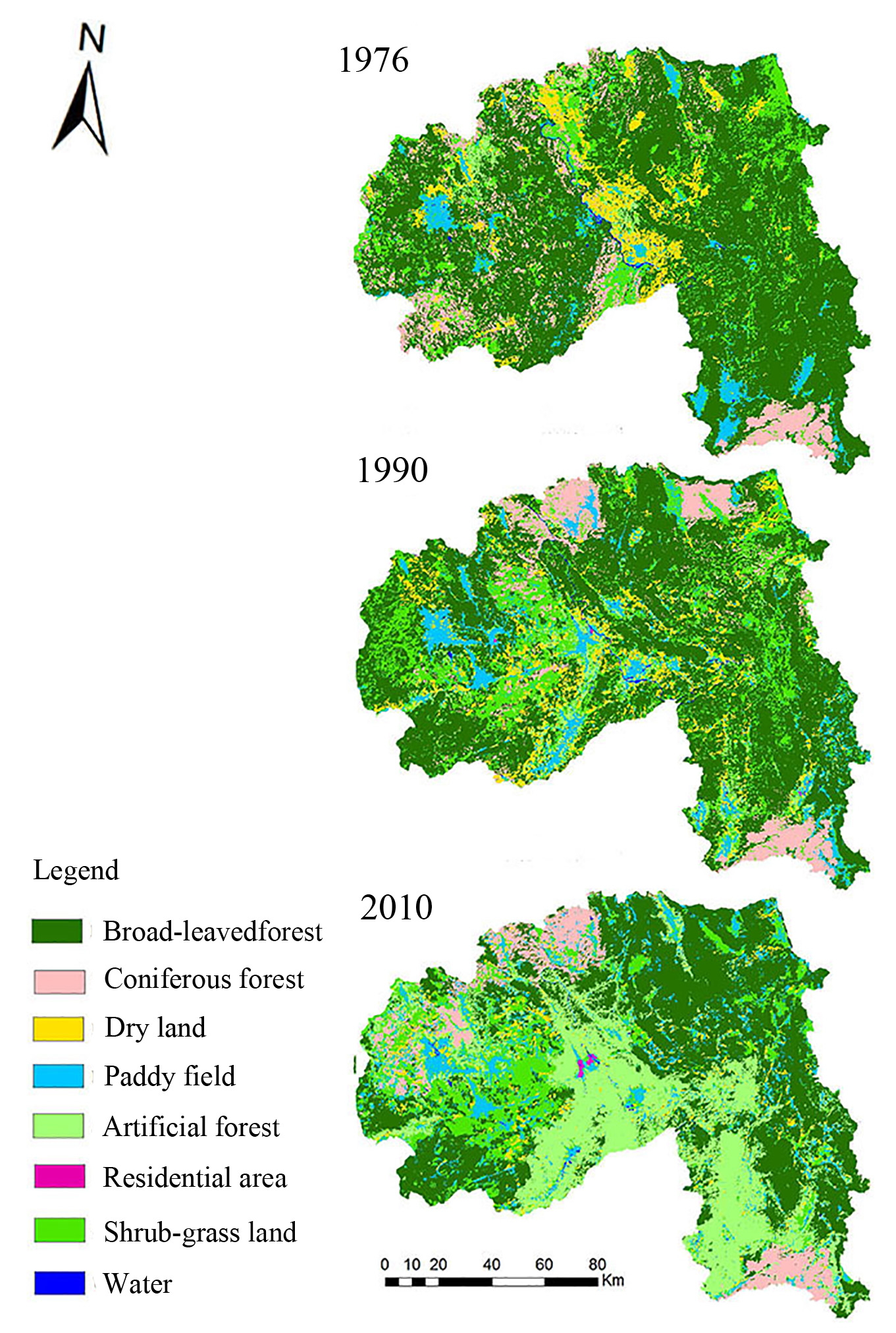

Supplement: S1 Fig — (TIF) [file pone.0189368.s003.tif]

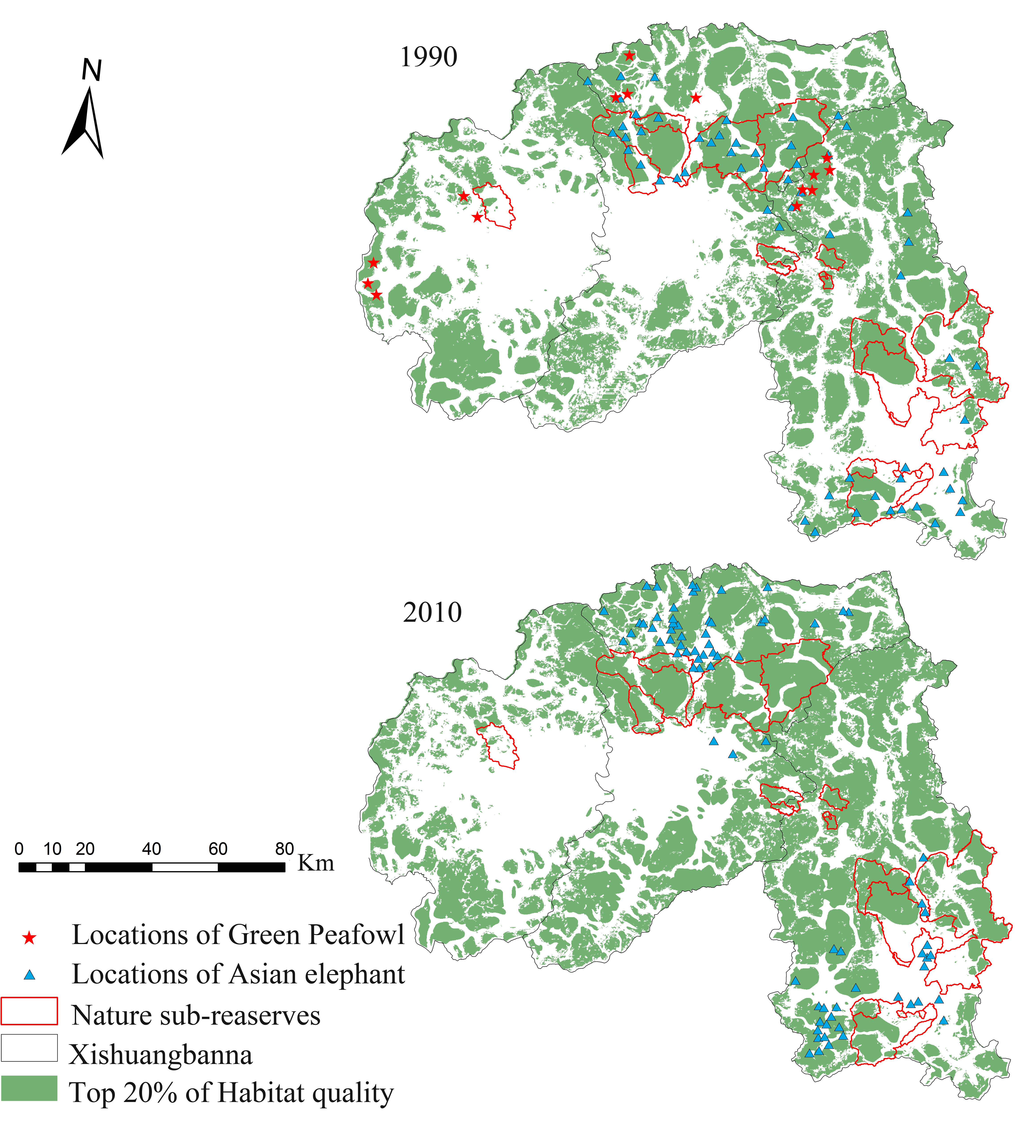

Supplement: S2 Fig — (TIF) [file pone.0189368.s004.tif]
